# A Meta-Model to Predict the Drag Coefficient of a Particle Translating in Viscoelastic Fluids: A Machine Learning Approach

**DOI:** 10.3390/polym14030430

**Published:** 2022-01-21

**Authors:** Salah A. Faroughi, Ana I. Roriz, Célio Fernandes

**Affiliations:** 1 Geo-Intelligence Laboratory, Ingram School of Engineering, Texas State University, San Marcos, TX 78666, USA; 2 Department of Polymer Engineering, Institute for Polymers and Composites (IPC), Campus of Azurém, Engineering School of the University of Minho, 4800-058 Guimarães, Portugal; b12374@dep.uminho.pt (A.I.R.); cbpf@dep.uminho.pt (C.F.)

**Keywords:** machine learning, deep learning, stacked learning, viscoelastic flows, Oldroyd-B fluid, Giesekus fluid, sphere drag coefficient

## Abstract

This study presents a framework based on Machine Learning (ML) models to predict the drag coefficient of a spherical particle translating in viscoelastic fluids. For the purpose of training and testing the ML models, two datasets were generated using direct numerical simulations (DNSs) for the viscoelastic unbounded flow of Oldroyd-B (*OB-set* containing 12,120 data points) and Giesekus (*GI-set* containing 4950 data points) fluids past a spherical particle. The kinematic input features were selected to be Reynolds number, 0<Re≤50, Weissenberg number, 0≤Wi≤10, polymeric retardation ratio, 0<ζ<1, and shear thinning mobility parameter, 0<α<1. The ML models, specifically Random Forest (RF), Deep Neural Network (DNN) and Extreme Gradient Boosting (XGBoost), were all trained, validated, and tested, and their best architecture was obtained using a 10-Fold cross-validation method. All the ML models presented remarkable accuracy on these datasets; however the XGBoost model resulted in the highest $R^{2}$ and the lowest root mean square error (RMSE) and mean absolute percentage error (MAPE) measures. Additionally, a blind dataset was generated using DNSs, where the input feature coverage was outside the scope of the training set or interpolated within the training sets. The ML models were tested against this blind dataset, to further assess their generalization capability. The DNN model achieved the highest $R^{2}$ and the lowest RMSE and MAPE measures when inferred on this blind dataset. Finally, we developed a meta-model using stacking technique to ensemble RF, XGBoost and DNN models and output a prediction based on the individual learner’s predictions and a DNN meta-regressor. The meta-model consistently outperformed the individual models on all datasets.

## 1. Introduction

The flow of particle-laden complex fluids has been the centerpiece of many well-documented experimental, theoretical, and numerical approaches [1,2,3,4]. These fluids are non-Newtonian in character showing shear thinning, shear thickening, viscoplastic, time-dependent and viscoelastic behaviors under different flow conditions. Resolving the dynamics of particles within these fluids is extremely challenging both experimentally and computationally, especially when the matrix fluid is viscoelastic, e.g., a polymer solution or polymer melt [4,5,6,7].

Characterizing the dynamics of particles in complex fluids under different flow and/or environmental conditions requires comprehensive experimentation and simulation tools to resolve the nonlinear interplay of multiple physical variables, flow parameters and many-body interactions [8]. This is computationally expensive even when using high performance computing resources with robust parallelized algorithms [4]. In recent decades, many physics-based numerical models have been proposed to model complex fluids, see the review by Maxey [9]. These approaches are limited to specific conditions and cannot be practically applied to large-scale engineering applications where hundreds or millions of particles are suspended, e.g., blood and other biological fluids, hydraulic fracturing, cementing, etc. [10,11]. In addition to the computing power, the insufficiency of comprehensive physics-based constitutive models to describe a broad range of complexities involved with such fluids, their use in realistic conditions encounter severe uncertainties or limitations. Therefore, the wealth of existing domain knowledge and scientific capabilities in this field need to be complemented with evolving technologies such as Machine Learning (ML) and Deep Learning (DL) to accelerate fundamental and applied research and close knowledge and computational gaps. For example, for a time intensive conventional computational model, both inner-loop (i.e., forward simulations) and the outer-loop (optimization and data assimilation) can be improved using the adaptivity and acceleration of ML- or DL-based models [12,13].

One main challenge for the application of ML and DL in the field of complex fluids is the lack of datasets. This challenge can be resolved by an in-line integration of traditional (e.g., CFD) and DL-based modeling, so-called a physics-informed DL, physics-guided DL, or digital-twin technique [14]. The ML or DL algorithms can be trained and integrated with traditional physics-based forward modeling to predict the flow dynamics under different conditions at a reduced computational cost. The latter is done by learning the solutions for ordinary and partial differential equations governing the system [13] or learning the closure laws for the pertinent physics, e.g., lift and drag forces, turbulence models, etc.

Such integration can be explored for the Eulerian-Lagrangian multi-phase model [6,15], as one of the main computational methods to resolve the flow of complex fluids. The ML/DL integration can drastically increase the robustness of this numerical algorithm that integrates the presence of multiple non-Brownian particles as the discrete material phase embedded in a viscoelastic fluid treated as a continuum phase. In particular, the momentum-exchange model, including drag, lift, hindrance, and retardation closure laws to couple the constituents [6,16,17], can be replaced with ML or DL models. In this approach, the fundamental goal is to provide reasonably accurate data-driven predictions that substitute expensive computational steps. These data-driven models learn the multitude of coupling within the complex fluids at the particle-level and enable accurate simulations of complex fluids at larger length and time scales.

In the present contribution, we propose to take the first step and complement the Eulerian-Lagrangian multi-phase approach with a data-driven drag model for the translation of a spherical particle in constant viscosity and shear thinning matrix-based viscoelastic fluids. In a constant viscosity elastic fluid, the drag coefficient decreases at low levels of elasticity, and increases at high elasticity due to the large elastic stresses developing on the surface and on the wake of the particles [1,18,19,20]. When the shear thinning effect is added, the drag coefficient decreases as elasticity increases. Recently, Faroughi et al. [6] developed a closure drag model for a single spherical particle translating through constant viscosity elastic fluids described by the Oldroyd-B constitutive equation. However, due to strong interaction of elasticity (e.g., high Weissenberg number) and kinematic parameters (shear thinning and thickening), a general solution for this problem that can integrate all dimensions of the data and perform well over a wide range of parameters, is still missing and cannot be formed using traditional approaches.

Accordingly, the present contribution is undertaken to achieve two goals. First, we generate and condition comprehensive datasets capturing the dynamics of a spherical particle translating through constant viscosity and shear thinning viscoelastic fluids. This is done using direct numerical simulations (DNSs) following the work of Faroughi et al. [6]. The data are processed and labeled for a set of operational conditions to be consumed by supervised ML and DL methodologies. Second, we develop data-driven drag models by examining several ML-based regression methods trained, validated and tested on the generated datasets. The performance, accuracy and productivity of different models are thoroughly evaluated based on common statistical measures.

The paper is organized in the following manner: in Section 2 we present the governing equations describing the transient, incompressible and isothermal laminar flows of viscoelastic matrix-based fluids. We also present the physical system and computational domain used to generate the training datasets. In Section 3, the ML-based regression algorithms employed to predict the viscoelastic drag coefficient are described. Next, in Section 4, we present the complexity of the training datasets in detail and compare the performance of each ML model employed to learn the characteristics of these datasets. A meta-model, based on different ML models, is then trained to fully describe the datasets. Finally, in Section 5, we summarize the main conclusions of this work.

## 2. Underlying Physics

The conservation equations governing transient, incompressible and isothermal laminar flow of viscoelastic fluids are the continuity, momentum balance and constitutive equations. The continuity and momentum balance equations read as follows,

(1) ∇·(ρu)=0

(2) $$\frac{\partial (\rho u)}{\partial t}+\nabla \cdot \left(\rho uu\right)+\nabla \cdot \left(pI\right)-\nabla \cdot \tau =0$$

where ρ is the fluid density, u is the velocity vector, *t* is the time, *p* is the pressure, I is the identity tensor and τ is the total extra-stress tensor, which is split into solvent ($\tau_{S}$) and polymeric ($\tau_{P}$) contributions, such that $\tau =\tau_{S}+\tau_{P}$. These stress terms are obtained by the following constitutive equations,

(3) $$\tau_{S}=\eta_{S}\left(\nabla u+\nabla u^{T}\right)$$

(4) $$\lambda {\overset{▿}{\tau }}_{P}+\tau_{P}+\frac{\alpha \lambda }{\eta_{P}}\tau_{P}\cdot \tau_{P}=\eta_{P}\left(\nabla u+\nabla u^{T}\right)$$

where $\eta_{S}$ and $\eta_{P}$ are the solvent and polymeric viscosities, respectively, λ is the fluid relaxation time, α is the mobility parameter and $\overset{▿}{\tau }P$ indicates the upper-convective time derivative of the polymeric extra-stress tensor defined as,

(5) $$\begin{array}{c} {\overset{▿}{\tau }}_{P}\equiv \frac{\partial \tau_{P}}{\partial t}+u\cdot \nabla \tau_{P}-\tau_{P}\cdot \nabla u-\nabla u^{T}\cdot \tau_{P} \end{array}$$

Equation (4) is known as the Giesekus viscoelastic constitutive model [21]. For the particular case where the mobility parameter is zero, α=0, Equation (4) reduces to the well-known quasi-linear elastic dumbbell fluid, the Oldroyd-B fluid. When written in the continuum formulation, viscoelastic fluid flows are well known to introduce numerical convergence difficulties at high Weissenberg numbers. This is mainly related to the lack of sufficient resolution of the discretization methods to resolve the exponential growth of stresses near critical points as the Weissenberg number is incremented. To prevent this issue in the calculation of the polymeric extra-stress tensor components, we follow the log-conformation approach [22,23] implemented in the OpenFOAM [24] computational library.

**Figure 1:**
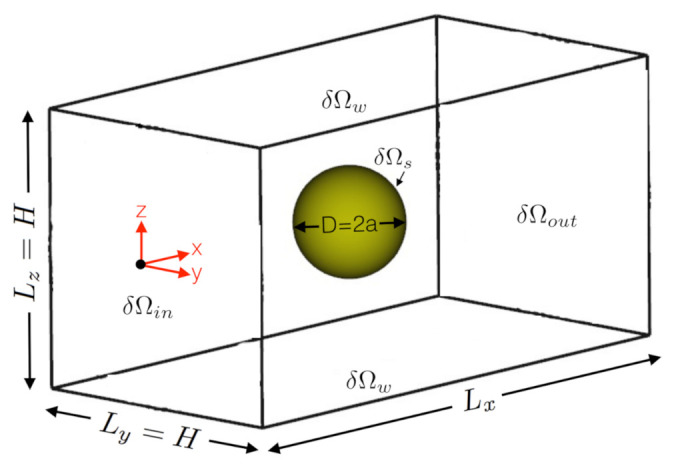
Schematic illustration of the computational domain (square duct) used to simulate the viscoelastic fluid flow past a sphere.

We use the above governing equations to perform an extensive set of DNSs and calculate the drag coefficient for a spherical particle translating in viscoelastic fluids. Figure 1 schematically illustrates the computational domain used in this study to simulate the unbounded viscoelastic flow around a sphere [6]. The domain size in flow direction, $L_{x}$, is considered larger than other dimensions to allow enough space for the polymer chains to be relaxed. Our numerical model and solver were comprehensively tested against this computational challenge (see, e.g., Fernandes et al. [25] and Faroughi et al. [6] for the case of an Oldroyd-B fluid flow around a sphere). All the numerical simulations were performed in parallel using several High-Performance Computing facilities (see Acknowledgments section). On a system with a 2.30 GHz AMD EPYC 7742 64-Core processor, the computational time for a single run was 12 ± 1 h.

For the present problem, we define the Reynolds and Weissenberg dimensionless numbers as follows,

(6) $$Re=\frac{2a\rho U}{\eta_{0}}$$

(7) $$Wi=\frac{\lambda U}{a}$$

where *U* is the inlet average fluid velocity and *a* is the sphere radius (D=2a is the sphere diameter). Additionally, the other dimensionless numbers considered in this work are the shear thinning mobility parameter α, and the polymeric viscosity ratio ζ. The latter is also known as the characteristic retardation ratio defined as,

(8) $$\zeta =\frac{\eta_{P}}{\eta_{S}+\eta_{P}}=\frac{\eta_{P}}{\eta_{0}}$$

where $\eta_{0}$ is the total fluid viscosity in the limit of vanishing shear rate and $\eta_{S}$ and $\eta_{P}$ are the solvent and polymeric contributions to the fluid viscosity, respectively.

Here, we carry out the calculations for the viscoelastic drag coefficient, $C_{D}$, using the surface integration of the total stress, $\tau =\tau_{P}+\tau_{S}$, and pressure field, *p*, on the surface of the sphere as,

(9) $$C_{D}=\frac{2}{\rho U^{2}A}\int_{\delta \Omega_{s}}\left(\tau_{P}+\tau_{S}-pI\right)\cdot n\cdot x\;dS$$

where *A* is the cross-sectional area of the sphere, n is the unit normal vector to the sphere surface, *S*, and x is the unit vector parallel to the flow direction. The results computed for the viscoelastic drag coefficient using Equation (9) are normalized by,

(10) $$\chi =\frac{C_{D}}{C_{D}\left(Wi\;=\;0\right)}$$

where χ is the viscoelastic drag coefficient correction factor [6].

## 3. Machine Learning Regression Algorithms

To relate the input features (i.e., a set of explanatory variables, Re, Wi, ζ and α) to the output features (i.e., the response variable, χ), different ML-based regression algorithms are employed in this work. These algorithms enable us to model multidimensional datasets which cannot be described using traditional techniques [6]. In the most basic form, the linear regression model explains a dependent variable *y* via a linear combination of the independent features, $x_{i}\;\left(i=1,\cdots ,n\right)$,

(11) $$\begin{array}{c} y=\beta_{0}+\beta_{1}x_{1}+\ldots +\beta_{n}x_{n}+\varepsilon \end{array}$$

where ε is an additive error and $\beta_{j}\;\left(j=0,\cdots ,n\right)$ are the coefficients of the features. Despite its simplicity, this model is widely used as a baseline and a tool to analytically study the independent variables and understand the significance of the input features. To achieve more accurate estimates and prevent the overfitting issue, we consider more sophisticated regression models such as ensemble decision tree algorithms (Random Forest and Extreme Gradient Boosting) and a Deep Neural Network (DNN). These methods possess their own challenges and should be applied with special care in scenarios where the training data are sparse [26].

The Random Forest (RF) is an ensemble learning technique that alleviates the overfitting issue and offers excellent performance within the scope of the training data [27]. In this approach, multiple decision trees are constructed at training time and the mean of the individual predictions is reported as the output of the ensemble method. At each candidate splitting within each tree model, a randomly selected subset of feature space is used. This trick has proven to be very effective and the resulting models are usually robust to the overfitting problem [28]. The RF models have emerged as a versatile and highly accurate regression methodology requiring little tuning while providing interpretable outputs. In summary, the RF algorithm includes (i) randomly select *n* subsamples, (ii) train the regression tree for each sample, and finally (iii) average all prediction results from all trees. This algorithm has 16 main hyperparameters as listed in Table 1, and the most important ones to tune are the n_estimators that represents the total number of trees in the forest, and Max_feature that represents the number of features to consider when looking for the best split. The selection of the feature for node splitting from a random set of features decreases the correlation between different trees and, thus, the average prediction of multiple regression trees is expected to have lower variance than individual regression trees [28].

The Extreme Gradient Boosting (XGBoost) algorithm proposed by Chen and Guestrin [29] is an improved algorithm of gradient boosting to recognize complex, nonlinear patterns inside datasets. One of the differences between XGBoost and RF models is related to the way the trees are built. In RF, trees are built independent of each other, but, in XGBoost, a new tree is added to complement the already built ones [30]. A prediction value $\left(y_{i}^{*}\right)$ from an ensemble model can be represented as,

(12) $$\begin{array}{c} y_{i}^{*}=h\left(x_{i}\right)=\sum_{k=1}^{K}f_{k}\left(x_{i}\right),\;i=1,\cdots ,N \end{array}$$

where $f_{k}$ is a regression tree, and $f_{k}\left(x_{i}\right)$ represents the score given by the *k*-th tree to the *i*-th observation in data. The goal in XGBoost is to minimize the regularized objective function expressed as [30],

(13) $$\begin{array}{c} L=\sum_{i=1}^{N}\Lambda \left(y_{i},y_{i}^{*}\right)+\sum_{k=1}^{K}\Omega \left(f_{k}\right) \end{array}$$

in order to choose functions $f_{k}$. Here, *N* is the number of observation (e.g., rows of data), Λ is the loss function which measures the accuracy and performance of the model in terms of its relationship between input ($x_{i}$) and output ($y_{i}$) features, and the penalty term Ω is included to prevent too large complexity of the model, being defined as [30]

(14) $$\begin{array}{c} \Omega \left(f_{k}\right)=\gamma T+\frac{1}{2}{\beta ||\omega ||}^{2} \end{array}$$

where γ and β are parameters controlling penalty for the number of leaves, *T*, and magnitude of leaf weights, ω, respectively. This penalty term makes XGBoost unique compared to general tree boosting methods. It has two main goals; (i) to prevent overfitting, and (ii) to simplify the end model produced by this algorithm. In addition to this regularized loss function, XGBoost is reinforced with two additional features that further prevent overfitting. First, the weights of each new tree can be scaled down reducing an impact of a single tree on the final score, which provides more room for next trees to improve the model [30]. The second feature is a column sampling working in a similar way as RF where each tree is built using only a column-wise sample from the training dataset [31]. The XGBoost algorithm has 24 main hyperparameters as listed in Table 1, divided in three categories: (a) general parameters as a guide to the overall functioning, (b) booster parameters as a guide to the individual booster at each step, and (c) learning task parameters as a guide to the optimization performance.

The Deep Neural Network (DNN) algorithms are one of the most commonly applied regression algorithms for stationary datasets [32]. The popular implementation is the multilayer perceptron (MLP), in which the architecture is optimized by iterating on various numbers of hidden neurons and layers that would lead to the best model with the highest accuracy on a dataset [33]. In MLP algorithm, the model is expressed as [34],

(15) $$\begin{array}{c} y=h\left(\varphi_{0}+\sum_{j=1}^{N}\varphi_{j}g\left(\sum_{i=1}^{M}\theta_{i}x_{i}\right)\right) \end{array}$$

where *N* and *M* represent the number of neurons in the hidden and input layers, respectively, *g* and *h* denote the transfer functions for the input layer and hidden layer, and the vector matrices of θ and φ represent the weight values for neurons in the input and hidden layers, respectively. A cost function is defined to measure the accuracy and performance of the model in terms of its relationship between input and output features. The objective in MLP is to minimize the cost function defined as [35],

(16) $$\begin{array}{c} Arg\;min:\frac{1}{2n}\sum_{i=1}^{n}{\left(h\left(x_{i}\right)-y_{i}\right)}^{2} \end{array}$$

where *n* is the number of samples and $h(x_{i})$ represents the model prediction. The batch gradient descent technique and stochastic gradient descent are the well-known optimization algorithms used to minimize the cost function [26]. These algorithms find the direction (gradient) necessary to minimize the cost function and often they are known as a hill-climbing approach [36]. It is important to note that a DNN model might have the highest accuracy in the training set obtained from multiple attempts, but it is prone to memorize the trend, noise, and detail in training set instead of intuitively understanding the trend in the dataset. Therefore, it loses the prediction capability. In order to avoid this, one may set a stoppage criteria for learning where the model tests its predictive capability on a validation set and stops training when validation accuracy departs from training accuracy. The DNN algorithm in total has 21 main hyperparameters as listed in Table 1, and the most important ones are hidden_layer_sizes and learning_rate [37].

## 4. Results and Discussion

**Figure 2:**
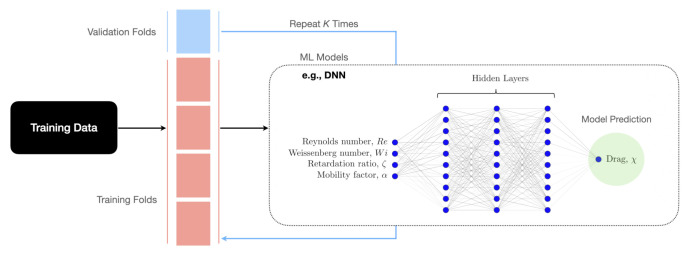
A summary of the training approach, input and output features to develop ML-based regression models (e.g., it is shown for a DNN model).

This section reports the processes taken to generate training datasets and develop ML models that predict the drag coefficient correction of a spherical particle translating in viscoelastic fluids described by the Oldroyd-B (constant viscosity fluids) and Giesekus (shear thinning fluids) constitutive equations. Figure 2 shows a summary of the inputs and output data considered in the development of the ML models described in Section 3. The input features are Reynolds number, Weissenberg number, retardation ratio, and mobility parameter, and the output variable is the drag coefficient normalized by the Newtonian value, i.e., χ as defined in Section 2. Figure 2 shows a schematic architecture for the DNN model.

### 4.1. Data Collection and Analysis for Oldroyd-B Fluids

For constant viscosity viscoelastic fluids [38], the relaxation time, λ, and retardation ratio, ζ, are the two important characteristics that define the viscoelastic behaviors. These fluids are generally modeled using the Oldroyd-B constitutive equation [39], and best represent very dilute polymer solutions at low Weissenberg number. Direct numerical simulations (DNSs), following the methodology implemented by Faroughi et al. [6] on the physical system elaborated in Section 2, were employed to generate the training dataset for the viscoelastic drag coefficient correction of a sphere translating in Oldroyd-B fluids (α=0). For that purpose, the range of the input features varied within 0<Re≤50, 0≤Wi≤10, and 0<ζ<1, which resulted in a total of 12,120 input values (hereafter we call this dataset *OB-set*). In addition to this dataset, we also generated a blind dataset, from a total of 60 DNSs, with an input feature coverage outside the scope or interpolated within the scope of *OB-set*. The blind dataset did not enter in the initial training, validation and testing phases and is used to scope the inference of the ML models beyond the limits of the training set, i.e., test the ML models’ generalization as described in Section 4.4.

**Figure 3:**
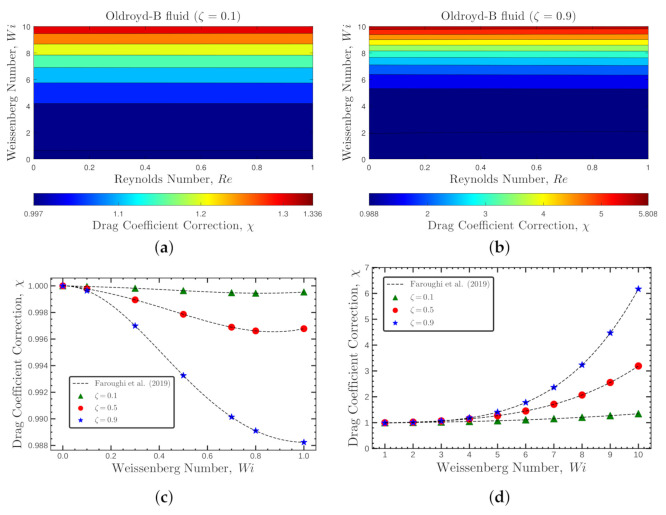
Contours of the viscoelastic drag coefficient correction, χ, for the Oldroyd-B fluid at 0<Re≤1, 0≤Wi≤10 and (**a**) ζ=0.1 and (**b**) ζ=0.9. Panels (**c**,**d**) show the variation of χ with Weissenberg numbers for different values of ζ at Re=1.

**Figure 4:**
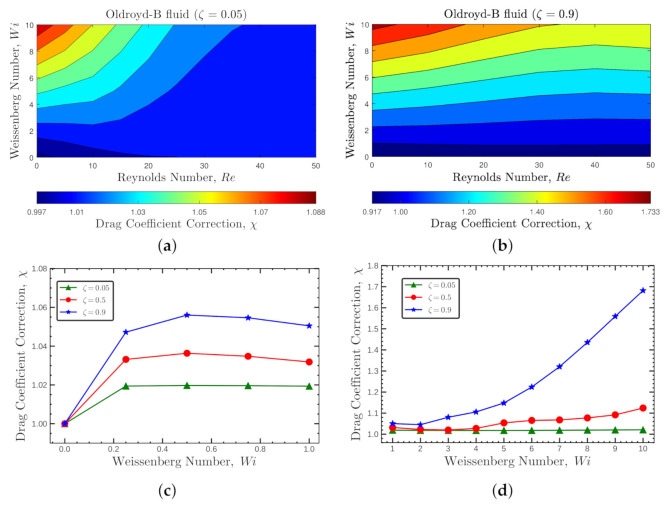
Contours of the viscoelastic drag coefficient correction, χ, for the Oldroyd-B fluid at 0<Re≤50, 0≤Wi≤10 and (**a**) ζ=0.05 and (**b**) ζ=0.9. Panels (**c**,**d**) show the variation of χ with Weissenberg numbers for different values of ζ at Re=50.

Figure 3 and Figure 4 show the flow characteristics associated with the *OB-set*. In Figure 3a,b, the contours of the viscoelastic drag coefficient correction, χ, are presented for Reynolds numbers 0<Re≤1 and Weissenberg numbers 0≤Wi≤10 at two polymeric retardation ratios ζ=0.1 and 0.9, respectively. The viscoelastic drag coefficient correction follows the same behavior for both ζ=0.1 and 0.9 across all considered Wi and Re numbers. As shown in Figure 3c,d, the viscoelastic drag coefficient correction slightly decreases at low Wi number, hits a minimum and then sharply increases with Wi number. The drag enhancement is more significant at higher ζ values (star blue symbols).

In Figure 4a,b, the contours of the viscoelastic drag coefficient correction, χ, are presented for Weissenberg numbers 0≤Wi≤10 and Reynolds numbers Re≤50 at two polymeric retardation ratios ζ=0.05 and 0.9, respectively. For both cases, the viscoelastic drag coefficient correction decreases with the increase of inertia and increases with Wi, being more noticeable for higher ζ. Figure 4c,d, show the behavior of χ at a fixed Reynolds number, Re=50. The viscoelastic drag coefficient correction increases up to Wi<0.2, then stays more or less constant up to Wi≈2, and then increases with Wi number, being this behavior more abrupt at higher retardation ratios.

**Figure 5:**
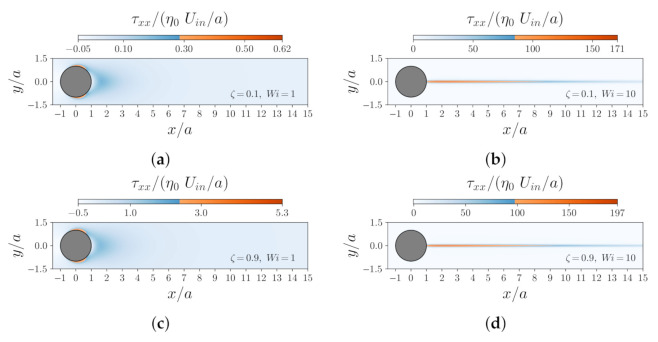
Contours of the dimensionless normal component of the polymeric extra-stress tensor $\tau_{xx}$ for the Oldroyd-B fluid at Re=1 for different values of ζ and Wi: (**a**) ζ=0.1 and Wi=1, (**b**) ζ=0.1 and Wi=10, (**c**) ζ=0.9 and Wi=1, and (**d**) ζ=0.9 and Wi=10.

Figure 5 shows the contours of the normal component of the dimensionless polymeric stress, $\tau_{xx}$, for different values of Wi and ζ at Re=1. The left column in Figure 5 shows the $\tau_{xx}$ contours at Wi=1, and the right column shows the same but at Wi=10. As expected, one observes that the magnitude of $\tau_{xx}$ increases as ζ increases. In addition, the location at which the maximum value of $\tau_{xx}$ occurs shifts from the top/bottom flow separation points to the wake of the particle as Wi increases. At Wi=10, for both ζ values, a long wake was also observed in the downstream region of the flow, where extensional flow dominates due to the significant effects of the flow elasticity.

Due to the similarity observed in the behavior of χ under different flow conditions (Re, Wi and ζ), the *OB-set* is an ideal dataset to be used in developing the building blocks for the ML-based models predicting the viscoelastic drag coefficient correction. However, the *OB-set* does not represents a large body of fluids that are encountered in nature or industrial applications. This issue is rectified in the next section using the shear thinning Giesekus constitutive equation [21] to augment the data.

### 4.2. Data Collection and Analysis for Giesekus Fluids

Most of the viscoelastic fluids show mid to strong shear thinning features. Shear thinning behavior leads to more complex and nonlinear dependencies at non-vanishing Weissenberg numbers, at which shear thinning effects become more pronounced. This behavior (neglected in the previous section) dramatically changes the behavior of the viscoelastic drag coefficient correction, χ. Therefore, the inference of the models trained based on the *OB-set* will certainly fail for shear thinning fluids. Hence, the *OB-set* must be augmented with data representing shear thinning fluids, or ML models developed based on the *OB-set* must be further trained to also account for shear thinning effects. Several viscoelastic constitutive models have been developed over the past few decades to model shear thinning fluids [40]. The Giesekus fluid is the one generally used to best represent the polymer molecules contribution to the momentum exchange in dilute to semi-dilute polymer solutions [41]. This model is based on a concept of configuration-dependent molecular mobility, and thus, the viscoelastic component of the polymeric stress tensor is represented by λ and ζ as well as the mobility parameter, α. The mobility parameter varies between zero and unity (practically 0.5 is the upper limit [41]) and accounts for the shear thinning behavior of the viscoelastic fluids.

We again used DNSs to generate the training dataset for the viscoelastic drag coefficient correction of a sphere translating in Giesekus fluids. A total of 4950 numerical simulations for the unbounded flow of the shear thinning viscoelastic Giesekus fluid past a sphere (using the physical system described in Section 2) were performed. Hereafter, we call this dataset *GI-set*. Simulations were conducted under a wide range of numbers for the input features, specifically 0<Re≤50, 0≤Wi≤10, 0<ζ<1, and 0<α<1. The end goal is to use *GI-set* to augment the *OB-set* and develop a ML-based meta-model that can be used by the scientific community to obtain a prediction of the dimensionless viscoelastic drag coefficient correction of a sphere translating in both Oldroyd-B and Giesekus fluids. We also generated a blind dataset using DNSs with flow features (Re, Wi, ζ and α) outside the ranges provided in the generation of *GI-set*. This dataset, consisting of 64 data points, is used to scope the accuracy of the ML models when inferred outside the limits of the training dataset, see Section 4.4.

**Figure 6:**
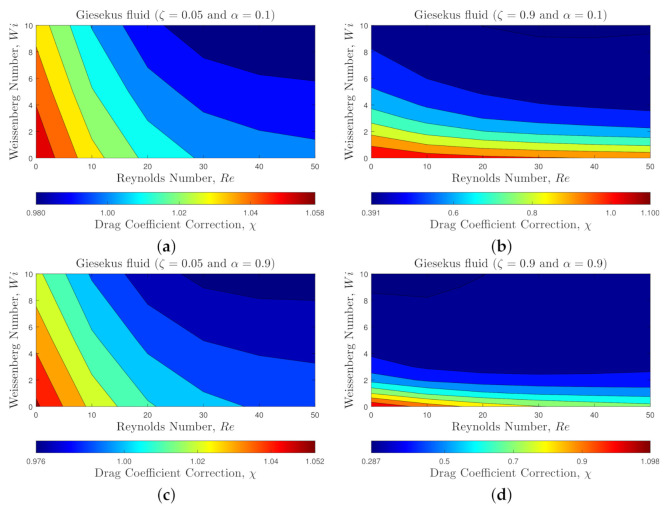
Contours of the viscoelastic drag coefficient correction, χ, for the shear thinning Giesekus viscoelastic fluid for 0<Re≤50, 0≤Wi≤10 and different values of α and ζ: (**a**) ζ=0.05 and α=0.1, (**b**) ζ=0.9 and α=0.1, (**c**) ζ=0.05 and α=0.9, and (**d**) ζ=0.9 and α=0.9.

**Figure 7:**
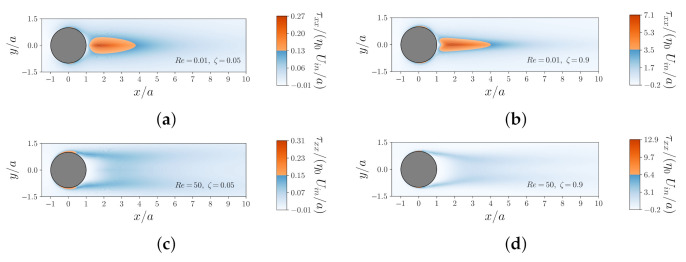
Contours of the dimensionless normal component of the polymeric extra-stress tensor $\tau_{xx}$ for the shear thinning Giesekus viscoelastic fluid at fixed Wi=5, α=0.1 and different values of Re and ζ: (**a**) Re=0.01 and ζ=0.05, (**b**) Re=0.01 and ζ=0.9, (**c**) Re=50 and ζ=0.05, and (**d**) Re=50 and ζ=0.9.

Figure 6 and Figure 7 present the flow characteristics associated with *GI-set*. Figure 6 shows the contours of the viscoelastic drag coefficient correction, χ, for 0<Re≤50 and 0≤Wi≤10 at polymeric retardation ratios ζ=0.05 and 0.9, and mobility parameters α=0.1 and 0.9. As shown in Figure 6, increasing inertia (i.e., Re number) leads to a reduction in the viscoelastic drag coefficient correction, similar to the behavior observed for the Oldroyd-B fluid. However, interestingly, increasing the elasticity of the flow (increasing the Wi number) results in a sharp reduction of the viscoelastic drag coefficient correction. The reduction is more pronounced at higher retardation ratio results. This behavior is totally different than what was observed for constant viscosity fluids, where the viscoelastic drag coefficient correction increases with Wi. Additionally, when the mobility parameter is increased (i.e., stronger shear thinning effect), it promotes the drag reduction even further as shown in Figure 6d.

**Figure 8:**
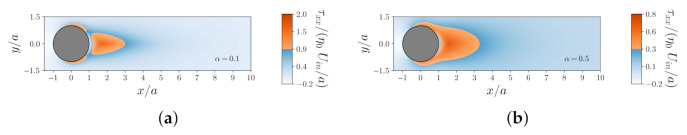
Contours of the dimensionless normal component of the polymeric extra-stress tensor $\tau_{xx}$ for the shear thinning Giesekus viscoelastic fluid at fixed Re=1, Wi=2, ζ=0.5 and different values of mobility parameter: (**a**) α=0.1 and (**b**) α=0.5.

To better illustrate the complexity of the flow and the effects of all flow features (Re, Wi, ζ and α), we compare the contours of the normal component of the dimensionless polymeric stress, $\tau_{xx}$, in Figure 7. These comparisons are shown for different values of Re (top line Re=0.01 and bottom line Re=50) and ζ (left column ζ=0.05 and right column ζ=0.9) at fixed Wi=5 and α=0.1. One observes that the magnitude of $\tau_{xx}$ increases as Re number increases. In addition, the change in the flow structure (i.e., flow separation and formation of symmetric eddies) due to Re number shifts around the location where the maximum of $\tau_{xx}$ occurs. Figure 7 also shows that an increase in the retardation ratio ζ promotes an elongated wake in the downstream of the flow. In Figure 8, we show the contours of $\tau_{xx}$ for the shear thinning Giesekus viscoelastic fluid for two different values of the mobility parameter, α=0.1 and 0.5, at fixed Re=1, Wi=2 and ζ=0.5. As expected and illustrated in Figure 8, increasing the mobility parameter decreases the stress overshoot on the surface as well as in the wake of the sphere, which in turn drastically hinders the enhancement of the viscoelastic drag coefficient correction due to elasticity (a behavior that was observed for Oldroyd-B fluids).

Figure 3, Figure 4, Figure 5, Figure 6, Figure 7 and Figure 8 collectively show the presence of a complex, multidimensional dynamics associated with a single spherical particle flowing through a viscoelastic fluid. All flow features (Re, Wi, ζ and α) strongly affect the flow fields and hence the viscoelastic drag coefficient correction (χ). These effects can be hardly decoupled to derive an analytical/empirical or semi-empirical expression for the viscoelastic drag coefficient correction prediction. A machine learning model, however, can be developed to learn these hidden features, in addition to features that are obvious to us in the data, to predict the viscoelastic drag coefficient of a spherical particle translating in an unbounded Oldroyd-B and Giesekus viscoelastic fluids.

### 4.3. ML Models Development

In this section, we leverage the *OB-set* and *GI-set* to train, validate and test the ML models discussed in Section 3. Based on these datasets, the design space for the input variables is defined as 0<Re≤50, 0≤Wi≤10, 0<ζ<1 and 0<α<1. First, a normalization stage is followed to restrict the input value range, which transforms the original input feature *x* to $\tilde{x}=\left(x-x_{min}\right)/\left(x_{max}-x_{min}\right)$. This is a common practice which speeds up learning and leads to faster convergence, especially for the DNN model. Next, we split each dataset to a training set (consisted of 80% of the data) and a test set (consisted of 20% of the data) that are bundled randomly. One of the primary objectives in this section is to improve the performance score, based on data patterns and observed evidence. To achieve this objective, the ML model architecture needs to be optimized by tuning a specific set of hyperparameters defined for each model (see Table 1 for a complete list of hyperparameters of the ML models considered in this study).

#### 4.3.1. Hyperparameter Tuning

Hyperparameter tuning relies more on experimental results than theory, and thus the best method to determine the optimal settings is to try many different combinations and evaluate the performance of each model. However, evaluating each model only on the training set can lead to overfitting (i.e., a model scores very well on the training set but performs poorly on the test set or blind dataset). Routinely, a subset of data from the training set, known as validation set, is reserved for this purpose. We adopt the K-Fold cross-validation (K-Fold CV) technique [42,43] to conduct hyperparameter tuning. In K-Fold CV technique, the training set is further split into K number of subsets, called folds, as schematically shown in Figure 2. The ML model is then iteratively fitted K times; each time, the training is done on K-1 of the folds and evaluation is done on the Kth fold (the validation set). At the very end of training, we average the performance on each of the folds to come up with final validation metrics for the model. The trained models each defined with specific hyperparameters are compared against each other, and the best one that offer the highest accuracy metrics is selected. In this study, unless otherwise stated, we apply 10-Fold CV, i.e., to assess a different set of hyperparameters, we split our training dataset into 10 folds and train and evaluate each model with selected hyperparameters 10 times. If we select X sets of hyperparameters using 10-Fold CV technique, which represents 10X training loops on the entire training dataset (e.g., X = 24 for XGBoost ML model). This process is thus computationally tedious. To facilitate that, K-Fold technique is coupled with RandomSearchCV algorithm to optimize selected hyperparameters [44]. This coupled approach tries random combinations within a range of values given for each parameter, with a defined number of iterations of random searches. The training time, using a workstation with 48 CPU cores and a NVIDIA RTX A8000 GPU, was on average 7 ± 0.5 h and 320 ± 8 h, respectively, for each iteration and all iterations required to perform hyperparameter tuning for a model.

To train and compare the performance of the ML models, the accuracy is evaluated based on three common statistical measures, $R^{2}$, RMSE, and MAPE. The latter, MAPE, represents the mean-absolute-value of the ratio of estimation errors to actual values. A lower MAPE value indicates that the predicted value is closer to the ground truth. The RMSE represents the root-mean-square error, which is also used to measure the differences between actual and predicted values by a model. The $R^{2}$ coefficient represents the fitness performance, i.e., higher values of $R^{2}$, with a max value of 1, are preferred. The mathematical expressions for $R^{2}$, RMSE and MAPE are as follows [45,46],

(17) $$\begin{array}{c} R^{2}=1-\frac{\sum_{i=1}^{n}{\left(y_{i}-y_{i}^{*}\right)}^{2}}{\sum_{i=1}^{n}{\left(y_{i}-{\bar{y}}_{i}\right)}^{2}}\\ \mathrm{RMSE}=\sqrt{\frac{1}{n}\sum_{i=1}^{n}{\left(y_{i}-y_{i}^{*}\right)}^{2}}\\ \mathrm{MAPE}=\frac{1}{n}\sum_{i=1}^{n}\left|\frac{y_{i}-y_{i}^{*}}{y_{i}}\right|\;\times \;100\% \end{array}$$

where *n* is the total number of observations, $y_{i}$ is the actual value, $y_{i}^{*}$ is the predicted value and ${\bar{y}}_{i}$ is the average of the actual values.

#### 4.3.2. Training and Testing

We first train, validate and test three different ML-based regression models to predict the drag coefficient correction of a single spherical particle translating through a viscoelastic fluid described by the Oldroyd-B constitutive equation (using the *OB-set*). We used 10-Fold CV approach in conjunction with RandomSearchCV algorithm for hyperparameters tuning, and employed the statistical measures given in Equation (17) to analyze the accuracy of the regression models. Table 2 reports the best set of hyperparameters (i.e., best architecture) obtained for each ML model. Notice that only hyperparameters that have been tuned are reported. These architectures, tuned by cross-validation technique on the *OB-set*, offer the best statistical measures for the predictions as reported in Table 3. The accuracy between real and predicted values is remarkable for all ML models as represented by the large $R^{2}$ values in Table 3. For the *OB-set*, XGBoost is the model that presents the best $R^{2}$ with the lowest values of RMSE and MAPE.

**Figure 9:**
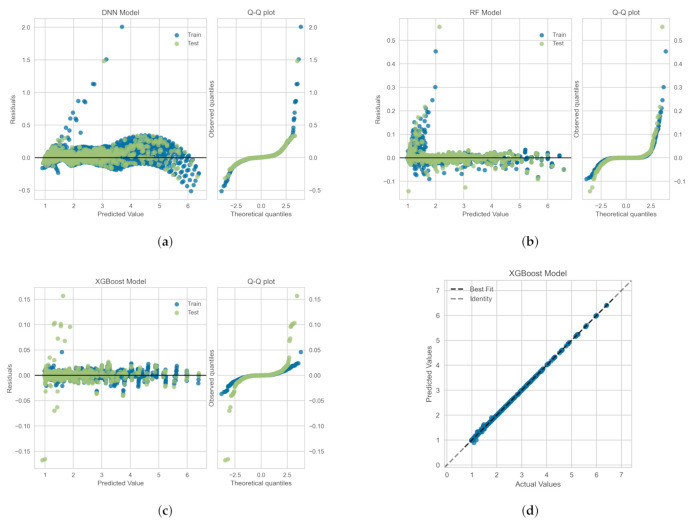
Residuals and quantile-quantile (Q-Q) plots obtained for the ML algorithms trained, validated and tested on the *OB-set*: (**a**) Neural Network, (**b**) Random Forrest, and (**c**) XGBoost models. Panel (**d**) shows the prediction error plot for XGBoost that yields the highest $R^{2}$ as reported in Table 3.

The residuals (or the prediction errors) and quantile-quantile (Q-Q) plots for the ML-based regression models trained, validated and tested on the *OB-set* are shown in Figure 9. The residuals are computed as the difference between the actual value (in the test set) and the values predicted by the optimized ML models. Figure 9 shows that the data points are mainly scattered around the horizontal axis and the calculated error is mainly distributed around zero. Figure 9 also shows Q-Q plots for each model in which the probability distributions for errors are compared for both train and test sets by plotting their quantiles against theoretical quantiles [47]. The theoretical quantiles on the *x*-axis represents normal distribution as the base distribution. This plot readily depicts whether or not the residuals (errors) are normally distributed. If points are close to the normal line, y=x, then residuals are assumed to be normally distributed. It can be seen that, for all models, most of the errors lies on y=0 line and data follow a heavy tail distribution [47]. Figure 9d illustrates the comparison between the actual and predicted values of the viscoelastic drag coefficient correction, χ, on the *OB-set* for the XGBoost model. As shown the best fit line coincides with the identity line, which corroborates the high $R^{2}$ value presented in Table 3.

The ML models trained on the *OB-set* fail, as expected, when inferred against a dataset generated for shear thinning fluids (e.g., even at α=0.1, which is just slightly outside the scope of the *OB-set* where α is set to zero). To accurately predict the drag coefficient correction of a spherical particle translating through a more realistic viscoelastic fluid, the trained models require further augmentation. For that purpose, three different approaches can be explored: (i) start training, validation and testing from scratch using a combination of the datasets developed for Oldroyd-B and Giesekus fluids (augmented *OB-set* and *GI-set*), (ii) use transfer learning technique [48] where models with knowledge gained on the *OB-set* are further reinforced using *GI-set*, or (iii) infuse physics in the models’ architecture using the constitutive fluid models as loss or activation function broadening the range over which the ML models are valid [49]. The latter approach is outside the scope of the current study and will be explored elsewhere. The accuracy obtained for the model derived by the second approach (i.e., transfer learning) was found to be significantly lower than the first approach when tested on the blind datasets. This is mainly due to the difficulty associated with transfer learning in decision tree ML models (Random Forest in particular where there is a limited capacity to accommodate local changes [50]). Thus, we adopted the first approach to develop ML-based models that satisfies both Oldroyd-B and Giesekus fluids. This approach is also challenging because datasets are not balanced. The weight of the *OB-set* (bigger dataset with 12,120 data points) is a lot larger than the *GI-set* (smaller dataset with 4950 data points), and consequently ML models will be more biased towards the *OB-set* (e.g., undermines the effects of α on the models’ predictability). To resolve this issue, we used synthetic minority over-sampling technique [51], SMOTE, which blends under-sampling of the majority set (*OB-set*) with a special form of over-sampling of the minority set (*GI-set*). In SMOTE, we synthesized elements for the minority set, based on the data that already exist. It works randomly by picking a point from the minority set and computing the k-nearest neighbors for this point. The synthetic points for minority set (*GI-set*) are placed between the chosen point and its neighbors. This process continues until we reach balanced states for both datasets, hereafter we call this dataset *SMOTE-set* containing 21,750 data points.

Again, we used 10-Fold CV approach in conjunction with the RandomSearchCV algorithm for the hyperparameters tuning of the ML models trained, validated and tested on *SMOTE-set*. Table 4 reports the best set of hyperparameters (i.e., best architecture) obtained for each one of the ML model employed in this work. These architectures offer the best statistical measures ($R^{2}$, RMSE and MAPE) for the ML-based regression models as reported in Table 5. The accuracy obtained for all ML models is acceptable as represented by the large $R^{2}$ and low RMSE values. For the *SMOTE-set*, again, the XGBoost model possesses the highest $R^{2}$, and the lowest values of RMSE and MAPE. This result is in agreement with the literature [44,52,53,54] and shows that the decision-tree models perform better than neural network models to learn hidden features on relatively midsize datasets. However, performing well on the test set still does not guarantee the accuracy of decision-tree-based regression models when inferred on blind datasets generated outside the scope of training set or interpolated within the training sets (see Section 4.4).

**Figure 10:**
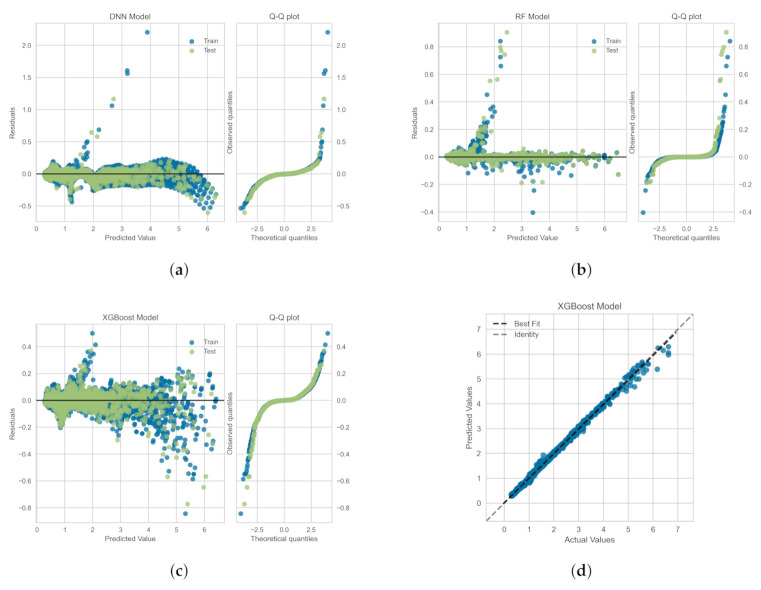
Residuals and quantile-quantile (Q-Q) plots obtained for the ML algorithms trained, validated and tested on *SMOTE-set*: (**a**) Neural Network, (**b**) Random Forrest, and (**c**) XGBoost models. Panel (**d**) shows the prediction error plot for XGBoost that yields the highest $R^{2}$ as reported in Table 5.

In Figure 10, we show the residuals and Q-Q plots for the ML models trained, validated and tested on *SMOTE-set*. The residuals are computed as the difference between the actual value (in the test set) and the values predicted by the optimized ML models. Figure 10 again shows that the data points are mainly scattered around the horizontal axis and the calculated error is mainly distributed around zero. The Q-Q plots for Random Forest and DNN models again depict that most of the errors lies on y=0 line, within the standard deviation range, and data follow a heavy tail distributions [47]. For the XGBoost model, the residuals are closer to the normal line, y=x, and thus, they follow a distribution closer to normal distribution. Figure 9d illustrates the comparison between the actual and predicted values of the viscoelastic drag coefficient correction, χ, for the *SMOTE-set* using the XGBoost model. This comparison corroborates the high $R^{2}$ values presented in Table 5 and the fact that the residual plot for XGBoost model is symmetric around y=0 as shown in Figure 9c.

### 4.4. Models Performance on Blind Datasets

To further evaluate the performance of the ML models trained on *SMOTE-set* in Section 4.3, we test them against blind datasets. We designed the blind datasets to have an input feature coverage outside the scope of training set or interpolated within the training sets. The blind datasets are generated using DNSs on the physical system described in Section 2 following the work of Faroughi et al. [6] for both Oldroyd-B and Giesekus fluids.

**Figure 11:**
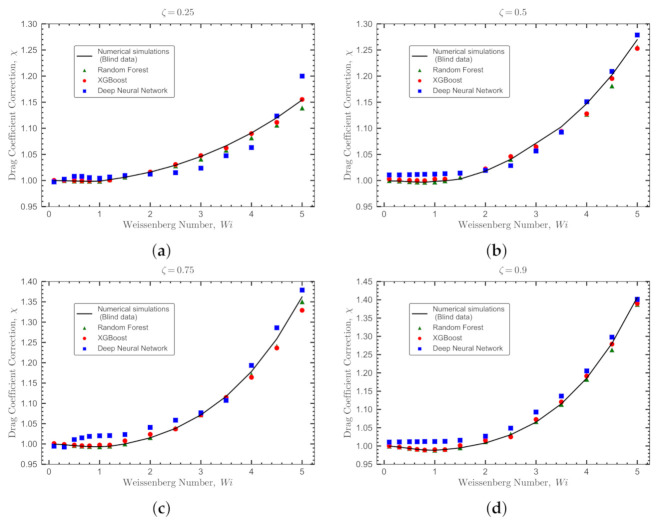
Validation of the ML models against the blind dataset generated for the Oldroyd-B fluid. The comparisons are between the DNSs (solid lines showing the real values for the viscoelastic drag coefficient correction) and the predicted values by the ML models. The comparisons are shown at Re=1 for different values of ζ: (**a**) ζ=0.25, (**b**) ζ=0.5, (**c**) ζ=0.75 and (**d**) ζ=0.9. The predictions obtained with the Deep Neural Network, Random Forest and XGBoost models are represented by square, triangle and circle symbols, respectively.

The blind dataset for the Oldroyd-B fluid is constructed with a total of 60 DNS runs using ζ={0.25,0.5,0.75,0.9}, Wi={0.1,0.3,0.5,0.65,0.8,1,1.2,1.5,2,2.5,3,3.5,4,4.5,5} at a constant Re=1. Figure 11 shows the comparisons between the real values for the viscoelastic drag coefficient correction (obtained by DNSs and represented with a solid line) and the values predicted by the ML models (represented by symbols). The ML models used in this comparison are those trained, validated and tested based on the *OB-set*. As depicted in Figure 11, all models perform very well to predict the blind dataset. The statistical measures for ML models to predict this blind dataset are reported in Table 6. The XGBoost model performs superior than other models and its predictions are in a very good agreement with the numerical results.

**Figure 12:**
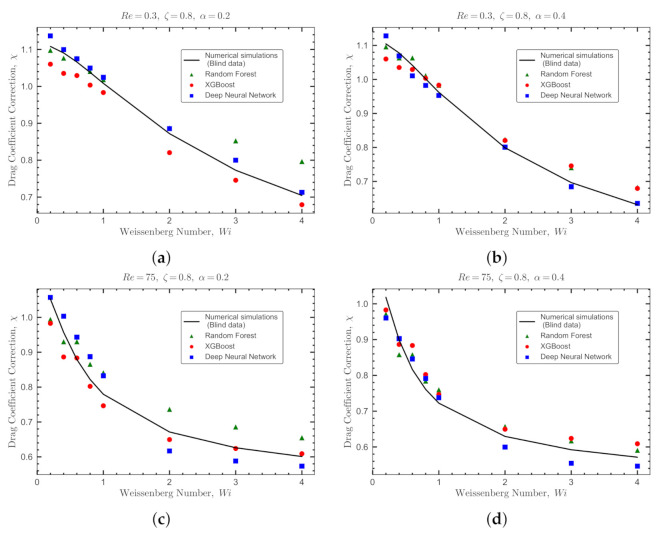
Validation of the ML models against blind datasets generated for Giesekus fluids. The comparisons are between the DNSs (solid lines showing the real values for the viscoelastic drag coefficient correction) and the predicted values by the ML models. The comparisons are shown at ζ=0.8 for different values of Re and α: (**a**) Re=0.3 and α=0.2, (**b**) Re=0.3 and α=0.4, (**c**) Re=75 and α=0.2, and (**d**) Re=75 and α=0.4. The predictions obtained with the Deep Neural Network, Random Forest and XGBoost models are represented by square, triangle and circle symbols, respectively.

The blind dataset for Giesekus fluids is constructed with a total of 64 DNS runs using Re={0.3,75}, ζ={0.15,0.8}, α={0.2,0.4}, and Wi={0.2,0.4,0.6,0.8,1,2,3,4}. Figure 12 shows the comparisons for two sample sets between the real values of the viscoelastic drag coefficient correction obtained by DNS (solid lines) and the predicted values by the ML models (symbols). The ML models used in this comparison are trained, validated and tested based on *SMOTE-set*. The statistical measures to predict this blind dataset are reported in Table 7. A sharp reduction in prediction performance is noticed for all models. This is due to two reasons: (i) the presence of values of Re which are out of the limits of the *SMOTE-set*, and (ii) the sparsity of data points in the combined dataset, i.e., *OB-set* and *GI-set*. Even using the SMOTE technique to balance the data sets (enforce the effect of high Re numbers and α), the ML models trained on *SMOTE-set* show a relatively poorer performance in predicting the blind dataset compared to the same models trained and tested on the *OB-set* (see Figure 11 and Table 6).

As shown in Figure 12, the DNN model performs slightly better than the decision tree models for both Re and α values. In general, ensemble decision tree models (e.g., XGBoost) are easy to train and prevent overfitting to a great extent [44,52,55]; however, they do not perform well in predicting sparse datasets where interpolation between input features is required. On the other hand, deep neural networks models are hard to train, but offer a better performance when inferred outside the scope of the training dataset or when interpolation between input features is needed [55,56]. Therefore, the DNN model provides a better potential for the generality of the model. In addition, for a ML model to be fully predictive under any new or unseen conditions (e.g., flow features), physics must complement the model. This can only be achieved using deep learning models, known as physics-based neural networks [57] or physics-guided neural networks [58] that mimic an infinitely deep model. Incorporating physics in DNN is essential in the field of particle-laden fluid flow, because it is not a data-oriented domain (i.e., large datasets can be hardly found). Developing true physics-based neural network is outside the scope of the current work, and it will be presented elsewhere. Here, to resolve this issue and provide a meta-model for the viscoelastic drag coefficient correction that can be coupled with Eulerian-Lagrangian algorithms [4], we use stacking technique [59]. This technique leverages the superiority of all developed ML models (i.e., the fact that each model performs better in a different section of the data), and is a very powerful method to increase the generality of the model in predicting unseen data.

### 4.5. Model Ensembling

In this section, we leverage stacking which is an ensemble learning technique to combine multiple ML-based regression models via a meta-regressor. The objective is to develop a meta-model with high accuracy when predicting the drag coefficient for a particle translating in viscoelastic fluids. In previous sections, we showed that different ML models perform better on different sections of the data when inferred against blind datasets. For example, the XGBoost performs better on the Oldroyd-B blind dataset (see Table 6), and the DNN model performs better on the Giesekus blind dataset (see Table 7). The hypothesis here is to leverage the superiority of all developed ML models (i.e., decision tree models to prevent overfitting and DNN model to learn complicated features in a sparse dataset) and increase the generality of the model in predicting unseen data.

**Figure 13:**
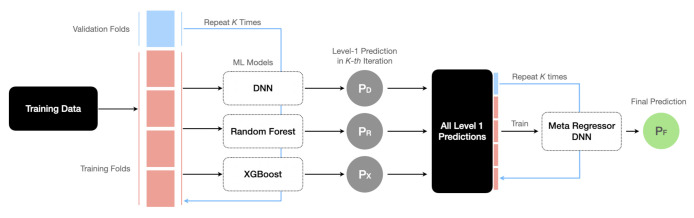
A schematic architecture for the meta-model to predict the viscoelastic drag coefficient using the stacking technique ensembling three optimized ML models and a meta-regressor.

A schematic architecture for the stack model is shown in Figure 13. We first use the ML models trained on *SMOTE-set* (with their best architectures found in the previous section) to provide the level-1 predictions. These predictions are then provided as input features to the second-level regressor, which is a DNN meta-regressor. The hyperparameters for DNN meta-regressor are also tuned again using the 10-Fold CV approach in conjunction with the RandomSearchCV algorithm, similar to other models. The stack model is trained, validated and tested on *SMOTE-set*. The optimized architecture obtained for the DNN meta-regressor is reported in Table 8. This meta-model developed using stacking generalizes better and provides more accurate predictions on unseen data when compared to the performance of the individual models. One example comparison is reported in Table 9. As reported, the $R^{2}$ value for the meta-model increased to 0.9472 from 0.9013, which was previously obtained for the DNN model, as the best model in Section 4.4 to predict the blind datasets.

**Figure 14:**
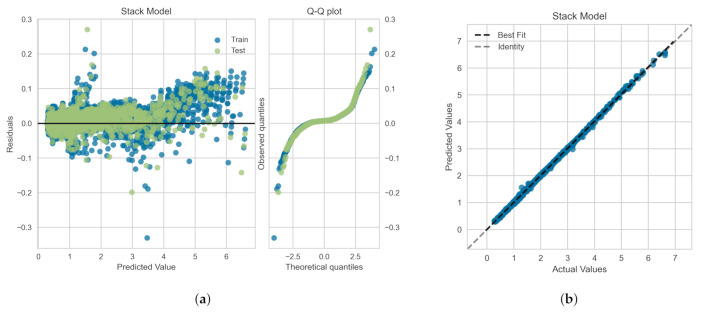
(**a**) Residuals and quantile-quantile (Q-Q) plots and (**b**) prediction error plot obtained for the meta-model shown in Figure 13 and trained, validated and tested on *SMOTE-set*. The statistical measures are reported in Table 9.

In Figure 14, we show the residuals and Q-Q plots for the meta-model trained, validated and tested on *SMOTE-set*. Figure 14a shows that the data points are mainly scattered around the horizontal axis and the calculated error is mainly distributed around zero. The Q-Q plot for meta-model depicts that most of the errors lies closer to y=x line within the standard deviation range, and thus the data follow a distribution closer to normal distribution [47]. Figure 14b illustrates the comparison between the actual and predicted values of the viscoelastic drag coefficient correction using the meta-model. The very good agreement between the meta-model predictions and the actual values corroborates the high $R^{2}$ values presented in Table 9 and the fact that the residual plot for meta-model is relatively symmetric around y=0 as shown in Figure 14a.

**Figure 15:**
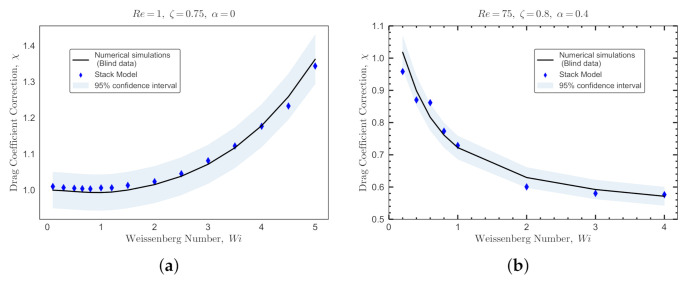
Performance of the meta-model (stack model) against the blind datasets generated for Oldroyd-B and Giesekus fluids. The blind datasets are shown using solid lines for the flow of an Oldroyd-B fluid past a sphere at (**a**) Re=1, ζ=0.75 and α=0; and for the flow of a Giesekus fluid past a sphere at (**b**) Re=75, ζ=0.8 and α=0.4. The predictions obtained with the meta-model are represented by diamond symbols. A 95% confidence interval region for the predictions is also shown.

Figure 15 shows the performance of the meta-model against the blind datasets generated for Oldroyd-B and Giesekus viscoelastic fluids. The blind datasets are shown using solid lines for the flow of an Oldroyd-B fluid past a sphere at Re=1, ζ=0.75 and α=0, and for the flow of a Giesekus fluid past a sphere at Re=75, ζ=0.8 and α=0.4. The predictions obtained with the meta-model are represented by diamond symbols in Figure 15a,b. A 95% confidence interval region for the meta-model predictions is also illustrated. These comparisons and the statistical measures reported in Table 9 collectively show that the meta-model consistently outperforms the individual decision tree ML models as well as the DNN model on all unseen datasets.

This meta-model alongside the training datasets (*OB-set* and *GI-set*) are packaged and published with this paper, as supplementary materials. The viscoelastic fluid dynamics community can leverage this meta-model in their simulations and/or leverage the data to train new data-driven models.

## 5. Conclusions

This study presents a framework to predict the drag coefficient of a spherical particle translating in viscoelastic fluids. To this end, continuum simulations and Machine Learning (ML) models were employed to generate a data-driven meta-model. We first generated two datasets using direct numerical simulations; the *OB-set* (the dataset for the Oldroyd-B fluid) and the *GI-set* (the dataset for the Giesekus fluid) that include a total of 12,120 and 4950 data points, respectively. The kinematic input features were selected to be Reynolds number, 0<Re≤50, Weissenberg number, 0≤Wi≤10, polymeric retardation ratio, 0<ζ<1, and shear thinning mobility parameter, 0<α<1. Three ML regression models, Random Forest (RF), Deep Neural Network (DNN) and Extreme Gradient Boosting (XGBoost), were employed to predict the drag coefficient enhancement or reduction due to the fluids’ elasticity and shear thinning effects. The ML models were all trained, validated, and tested on the *OB-set* and *SMOTE-set* (a balanced dataset combining the *OB-set* and *GI-set*), and their best architecture (i.e., tuned hyperparameters) were obtained using a 10-Fold cross-validation method. All the ML models presented remarkable accuracy when trained and inferred on these datasets; however the XGBoost model resulted in the highest $R^{2}$ and lowest RMSE and MAPE measures.

The trained ML models were also tested against a blind dataset where the input features coverage was outside the scope of the training set or interpolated within the training sets. A total of 124 data points were generated using DNSs for both Oldroyd-B and Giesekus fluids. The predictions obtained with the DNN model achieved the highest $R^{2}$ and lowest RMSE and MAPE measures when inferred on the blind test dataset. To leverage the power of all models (decision tree models to prevent overfitting and DNN model to learn complicated features), we developed a meta-model using stacking technique. The meta-model ensembles RF, XGBoost, and DNN models and outputs a prediction based on the individual learner’s predictions and a DNN meta-regressor. The meta-learner model consistently outperformed the individual decision tree and DNN models on all datasets.

## Figures and Tables

**Table 1:** Tunable hyperparameters in different machine learning regression models applied in this study.

Model	Hyperparameters	Total Number
Random Forest	Bootstrap, criterion, max_depth, max_features, max_leaf_nodes, min_impurity_decrease, min_impurity_split, min_samples_leaf, min_samples_split, min_weight_fraction_leaf, n_estimators, n_jobs, oob_score, random_state, verbose, warm_start	16
XGBoost	base_score, booster, colsample_bylevel, colsample_bynode, colsample_bytree, gamma, importance_type, learning_rate, max_delta_step, max_depth, min_child_weight, missing, n_estimators, n_jobs, nthread, objective, random_state, reg_alpha, reg_lambda, scale_pos_weight, seed, silent, subsample, verbosity	24
DNN	activation, alpha, batch_size, beta_1, beta_2, early_stopping, epsilon, hidden_layer_sizes, learning_rate, learning_rate_init, max_iter, momentum, n_iter_no_change, nesterovs_momentum, power_t, random_state, shuffle, solver, tol, validation_fraction, verbose, warm_start	21

**Table 2:** Optimized hyperparameters for different ML-based regression models trained, validated and tested on the *OB-set*.

Model	Hyperparameter Value
Random Forest	bootstrap = True, criterion = mse, max_depth = 110, max_features = auto, min_samples_leaf = 3, min_samples_split = 5, n_estimators = 800
XGBoost	colsample_bynode = 0.8, colsample_bytree = 0.8, learning_rate = 0.1, max_depth = 15, n_estimators = 300, objective = reg:gamma, reg_alpha = 1.2, reg_lambda = 1.3, subsample = 0.7
DNN	activation = relu, alpha = 0.0001, hidden_layer_sizes = (40, 20, 10), learning_rate = adaptive, max_iter = 8000, momentum = 0.9, n_iter_no_change = 10, solver = adam, tol = 0.0001

**Table 3:** Optimal statistical measures for different ML-based regression models trained, validated and tested on the *OB-set*.

	RF	XGBoost	DNN
*R* ^2^	0.9991	**0.9995**	0.9989
RMSE	0.0228	**0.0134**	0.0451
MAPE	0.0048	**0.0012**	0.0145

**Table 4:** Optimized hyperparameters for the different ML-based regression models trained, validated and tested on *SMOTE-set*.

Model	Hyperparameter Value
Random Forest	bootstrap = True, criterion = mse, max_depth = 45, max_features = auto, min_samples_leaf = 3, min_samples_split = 5, n_estimators = 950
XGBoost	colsample_bynode = 1, colsample_bytree = 0.8, learning_rate = 0.2, max_depth = 5, n_estimators = 400, objective = reg:gamma, reg_alpha = 1.2, reg_lambda = 1.3, subsample = 0.7
DNN	activation = relu, alpha = 0.0001, hidden_layer_sizes = (120, 50, 20, 3), learning_rate = adaptive, max_iter = 10,000, momentum = 0.95, n_iter_no_change = 15, solver = adam, tol = 0.0001

**Table 5:** Optimal statistical measures for different ML-based regression models trained, validated and tested on *SMOTE-set*.

	RF	XGBoost	DNN
*R* ^2^	0.9965	**0.9967**	0.9945
RMSE	0.0388	**0.0378**	0.0463
MAPE	0.0131	**0.0127**	0.0216

**Table 6:** Statistical measures for different ML-based regression models tested against the blind dataset provided for Oldroyd-B fluids.

	RF	XGBoost	DNN
*R* ^2^	0.9926	**0.9931**	0.9621
RMSE	0.0085	**0.0074**	0.0173
MAPE	0.051	**0.0042**	0.0139

**Table 7:** Statistical measures for ML-based models tested against the blind dataset provided for Oldroyd-B and Giesekus fluids.

	RF	XGBoost	DNN
*R* ^2^	0.8664	0.8566	**0.9013**
RMSE	0.0495	0.0516	**0.0428**
MAPE	0.0326	0.0341	**0.0305**

**Table 8:** Optimized hyperparameters for the DNN meta-regressor trained, validated and tested on *SMOTE-set*.

Model	Hyperparameter Value
DNN Meta-Regressor	activation = relu, alpha = 0.0001, hidden_layer_sizes = (20, 40, 10), learning_rate = adaptive, max_iter = 10,000, momentum = 0.95, n_iter_no_change = 15, solver = adam, tol = 0.0001

**Table 9:** Comparison of the statistical measures for the performance of the meta-model and DNN model against the *SMOTE-set* and blind datasets.

	Meta-Model		DNN Model	
	SMOTE-Set	Blind Set	SMOTE-Set	Blind Set
*R* ^2^	**0.9993**	**0.9472**	0.9945	0.9013
RMSE	**0.0178**	**0.0313**	0.0463	0.0428
MAPE	**0.0103**	**0.0209**	0.0216	0.0305

## Data Availability

Not applicable.

## Supplementary Materials

The following supporting information can be downloaded at: https://www.mdpi.com/article/10.3390/polym14030430/s1.
